# Diversity and biofilm-production ability among isolates of *Escherichia coli* phylogroup D belonging to ST69, ST393 and ST405 clonal groups

**DOI:** 10.1186/1471-2180-13-144

**Published:** 2013-06-21

**Authors:** Ângela Novais, Claudia Vuotto, João Pires, Carolina Montenegro, Gianfranco Donelli, Teresa M Coque, Luísa Peixe

**Affiliations:** 1 Departamento de Microbiologia, REQUIMTE, Faculdade Farmácia Universidade Porto, Rua Jorge Viterbo Ferreira n° 228, 4050–313 Porto, Porto, Portugal; 2 Microbial Biofilm Laboratory (LABIM), IRCCS Fondazione Santa Lucia, Rome, Italy; 3 Servicio de Microbiología and CIBER en Epidemiología y Salud Pública (CIBERESP), Instituto Ramón y Cajal de Investigación Sanitaria (IRYCIS), Hospital Universitario Ramón y Cajal, Madrid, Spain

**Keywords:** ExPEC, High-risk clones, ESBL, Virulence, Adhesion, Biofilm

## Abstract

**Background:**

Phylogenetic group D *Escherichia coli* clones (ST69, ST393, ST405) are increasingly reported as multidrug resistant strains causing extra-intestinal infections. We aim to characterize inter- and intraclonal diversity of a broad sample (isolates from different geographic locations and origins with variable antibiotic resistance profiles, 1980-2010) and their ability to adhere and form biofilm by both a modified quantitative biofilm producing assay and Field Emission Scanning Electron Microscopy (FESEM).

**Results:**

High virulence scores were observed among ST69 (median 14/range 9–15) and ST393 (median 14/range 8–15) clones, particularly enriched in *pap* alleles, *iha*, *kpsMTII*-K5 and *ompT*, in contrast with ST405 (median 6/range 2–14) isolates, exhibiting frequently *fyuA*, *malX* and *traT*. All ST69 and ST393 and only two ST405 isolates were classified as ExPEC. Biofilm production was detected in two non-clinical ST69 and three ST393 isolates from different origins showing variable virulence profiles. Within each clonal group, and despite the high diversity of PFGE-types observed, isolates from different countries and recovered over large periods of time were clustered in a few groups sharing common virulence gene profiles among ST69 (n = 10 isolates) and ST393 (n = 9 isolates) (*fimH*-*iha*-*iutA*-*kpsMTII*-*K5*-(*traT*)-*sat*-(*ompT*)-*papA*-*papEF*-*papGII*-*papC*) or ST405 (n = 6 isolates) (*fimH*-*traT*-*fyuA*-*malX*).

**Conclusions:**

This study highlights the circulation of highly transmissible ST69, ST393 and ST405 variants among different settings. Biofilm production seems not to be directly correlated with their epidemiological success.

## Background

Multidrug resistant *Escherichia coli* clones of the phylogenetic group D causing extraintestinal human infections are increasingly reported all over the world [1-4]. Among them, *E*. *coli* clonal groups D-ST69 (also recognized as clonal group A or CGA) and D-ST393 (also known as O15:K52:H1 clonal group) are widely spread among different hosts, often causing urinary tract infections (UTI) and conferring resistance to antibiotics [5-10]. Isolates belonging to the D-ST405 have been involved in the spread of genes encoding extended spectrum β-lactamases (ESBLs) (mainly CTX-M enzymes), cephamycinases (AmpC), carbapenemases (NDM) or methylases (AmrA, RmtB) [2,11-15]. Recent studies have reported variants of ST69, ST393 and ST405 among ESBL and non-ESBL-producing strains from specific locations [4,5,8,9,13,16]. The presence of genes possibly involved in biofilm production detected in some of these surveys (*fimH*, *papC*, *papG*, *fyuA* or *kpsMT II*) suggests the ability of these clones to adhere and form biofilm, which could be favouring their persistence; however, such ability has not been specifically evaluated [5,8,13,17,18]. Biofilm growing ability of bacteria is commonly assessed by a quantitative measure of their adherence to microtiter plates, although electron microscopy analyses provide more accurate information on the biofilm structure and presence of matrix [17-19].

In this study, we aim to characterize the intraclonal diversity of extraintestinal pathogenic *E*. *coli* (ExPEC) isolates from phylogenetic group D (ST69, ST393, ST405) isolated from different geographic locations and sources, and to assess their ability to adhere and form biofilm on abiotic surfaces in order to evidence a possible contribution of biofilm formation to their persistence and epidemicity.

## Methods

### Bacterial isolates

We analysed thirty-five *E*. *coli* isolates belonging to ST69 (n = 13), ST393 complex (10 ST393, 1 ST2321) and ST405 complex (10 ST405, 1 ST964) isolated from multiple sources and countries. They include either isolates associated with nosocomial or community outbreaks in different countries [2,4,9,12,20] or isolates collected from non-clinical sources from distinct countries and showing variable antibiotic resistance profiles, selected from published papers by the end of 2010. They were recovered from nosocomial (66%) and community-acquired infections (17%), healthy volunteers (8%), food products (6%) or animals (3%) and produced diverse ESBL or AmpC enzymes. Their epidemiological features are shown in Table 1. Isolates were taken as part of standard patient care and no ethical approval was required for their use.

**Table 1:** Epidemiological data and diversity among ST69, ST393 and ST405 clonal groups

**ST (N°)**	**Serotype**	**PFGE-type (Cluster)**	**Country (N° isolates)**	**Date**	**Origin**^**a**^	**Source (N° isolates)**	**ESBL / AmpC**	**Antibiotic resistance profile**^**b**^	**Virulence gene profile**^**b**^	**Reference**
69	O11, O73, O77	69_1 (I)	US (4), NW (1)	1999-2002	H	Urine (3), blood (2)		(Cm), Sm, Su, (Te), Tp, Ts	*fimH*,*iha*, *iutA*, *kpsMTII*, *K5*, *traT*, *sat*, (*ompT*), (*fyuA*), *papA*, *papEF*, *papGII*, *papC*,(*papGIII*), (*iroN*), (*iss*), (*ireA*)	[20,21]
69	O77	69_2 (I)	SP (1)	-	H	Urine	-	Sm, Su, Te, Tp, Ts	*fimH*, *iha*, *iutA*, *kpsMTII*, *K5*, *traT*, *sat*, *ompT*, *fyuA*, *papA*, *papEF*, *papGII*, *papC*, *papGIII*	[20]
69	-	69_3 (I)	BR (1)	-	H, C	-	-	(Ak), Cm, (Gm), (Km), (Nt), Sm, Su, (Tb), Te, Tp, Ts	*fimH*, *iha*, *iutA*, *kpsMTII*, *K5*, *traT*, *sat*, *ompT*, *fyuA*, *papA*, *papEF*, *papGII*, *papC*	[12,22]
			NW (1)	2006		Urine	CMY-2			
69	-	69_4 (I)	PT (1)	2007	H	Urine	-	Cp, Na, Sm, Su, Tp, Ts	*fimH*, *iha*, *iutA*, *kpsMTII*, *K5*, *traT*, *sat*, *ompT*, *papEF*, *iroN*, *iss*	This study
69	O17	69_5 (I)	US (1)	-	H	Blood	-	Cm, Sm, Su, Te, Tp, Ts	*fimH*, *iha*, *iutA*, *kpsMTII*, *K5*, *traT*, *sat*, *ompT*, *fyuA*, *papA*, *papEF*, *papGII*, *papC*, *papGIII*	[20]
69	-	69_6 (II)	PT (2)	2010	S	-	-	Km, Sm, Su, Te, Tp, Ts	*fimH*, *kpsMTII*, *K5*, *traT*, *ompT*, *papA*, *papEF*, *papC*, *papGIII*, *bmaE*, *gafD*, *iroN*, *iss*	[23]
69	-	69_7 (II)	NW (1)	2002	A	Poultry meat	-	Sm, Su, Te, Ts	*fimH*, *iutA*, *traT*, *ompT*, *papC*, *iroN*, *iss*, *tsh*, *ireA*	[21]
393	O15	NA^c^	US (1)	1980	H	-	-	-	*fimH*, *iha*, *iutA*, *kpsMTII*, *K5*, *sat*, *ompT*, *fyuA*, *papA*, *papEF*, *papGII*, *papC*, *papGIII*, *tsh*	[4]
393	O15	NA^d^	US (1)	1998	H	-	-	Cm, Gm, Km, Nt, Sm, Su, Tb, Te, Tp, Ts	*fimH*, *iha*, *iutA*, *kpsMTII*, *K5*, *sat*, *ompT*, *fyuA*, *papA*, *papEF*, *papGII*, *papC*	[4]
393	O15	NA^e^	US (1)	1999	H	-		Cp, Km, Na, Sm, Su, Te, Tp, Ts	*fimH*, *iha*, *iutA*, *kpsMTII*, *K5*, *sat*, *fyuA*, *papA*, *papEF*, *papGII*, *papC*, *tsh*, *papGI*	[4]
393	O15	NA^e^	KO (1)	2006-7	C	Urine	-	Cp, Gm, Km, Na, Nt, Sm, Su, Tb, Te, Tp, Ts	*fimH*, *iha*, *iutA*, *kpsMTII*, *K5*, *K1*, *traT*, *sat*, *ompT*, *fyuA*, *papA*, *papEF*, *papGII*, *papC*	[9]
393	O15	NA^e^	FR (1)	2006	F	Feces	-	Cm, Cp, Gm, Km, Na, Nt, Sm, Su, Tb, Te, Tp, Ts	*fimH*, *iha*, *iutA*, *kpsMTII*, *K5*, *traT*, *sat*, *ompT*, *papA*, *papEF*, *papGII*, *papC*, *iss*, *tsh*	[24]
393	O25	NA	FR (1)	2006	F	Feces	-	Cp, Na, Sm, Su, Te, Tp, Ts	*fimH*, *iha*, *iutA*, *kpsMTII*, *K5*, *sat*, *ompT*, *fyuA*, *papA*, *papEF*, *papGII*, *papC*, *iss*	[24]
393	O15	NA^e^	FR (1)	2006	F	Feces	-	Cm, Cp, Gm, Km, Nt, Sm, Su, Tb, Te, Ts	*fimH*, *iha*, *iutA*, *kpsMTII*, *K5*, *K1*, *traT*, *sat*, *fyuA*, *papA*, *papEF*, *papGII*, *papC*, *iss*, *tsh*	[24]
393	O15	NA	SP (1)	2002	C	Urine	CTX-M-14	Cp, Na, Sm, Su, Ts	*fimH*, *iha*, *iutA*, *kpsMTII*, *K5*, *sat*, *papA*, *papEF*, *papGII*, *papC*, *iss*, *tsh*	[25]
393	O15	NA^e^	KO (1)	2006-7	C	Urine	-	Cp, Km, Na, Sm, Su, Te, Tp, Ts	*fimH*, *iha*, *iutA*, *kpsMTII*, *K5*, *papEF*, *papGII*, *papC*	[9]
393	O15	NA^e^	NW (1)	2005	H	Urine	CMY-2	Cp, Km, Na, Nf, Sm, Su, Tp, Ts	*fimH*, *iutA*, *kpsMTII*, *K5*, *K1*, *ompT*, *fyuA*, *iss*, *tsh*	[12]
2321	O25	NA^e^	PT (1)	2008	H	Urine	TEM-like	Cp, Na, Sm, Su, Te, Tp, Ts	*fimH*, *iha*, *iutA*, *kpsMTII*, *K5*, *K1*, *sat*, *fyuA*, *papGII*, *papC*, *papGIII*, *iss*, *tsh*, *malX*, *iroN*	This study
405	-	405_1 (I)	SP (1), KU (1)^f^ NW (1)	2002-2004	H	Wound (1) Urine (1) Respiratory (1)	CTX-M-15 (2), CTX-M-3 (1)	(Cp), Cm, Gm, Km, Na, (Nt), Sm, Su, Tb, Te, Tp, Ts	*FimH*, (*iha*), *iutA*, (*kpsMTII*), *traT*, *sat*, (*malX*), *fyuA*, (*kpsMTIII*), (*iss*), (*tsh*)	[2,12]
405	-	405_2 (I)	NW (1)	2003	H	Wound	CTX-M-14	Cm, Km, Sm, Su, Te, Tp, Ts	*fimH*, *traT*, *malX*, *fyuA*, *kpsMTIII*	[12]
405	-	405_3 (I)	NW (1) PT (1) ^f^	2003-2006	H	Abcess (1) Urine (1)	CMY-2 (1) CTX-M-15 (1)	(Gm), (Km), Na, Sm, Su, (Tb), Te, Tp, Ts	*fimH*, (*iha*), *iutA*, (*kpsMTII*), *traT*, *malX*, *fyuA*, (*papEF*), (*papGIII*), (*iroN*), (*iss*), (*tsh*), (*ireA*)	[2,12]
405	-	405_4 (II)	SW (1) SP (1)	2000-2005	H, C	Urine (1) Blood (1)	CTX-M-15 TEM-24	(Cm), Cp, (Gm), Km, Na, (Nt), Sm, Su, Te, Tb, Tp, Ts	(*fimH*), (*iutA*), *traT*, *malX*, *fyuA*, *kpsMTIII*, (*iss*), (*tsh*)	[2,26]
405	-	405_5 (II)	SP (1)	2001	H	Wound	TEM-52	Cm, Cp, Gm, Km, Na, Sm, Su, Tb, Te, Ts	*traT*, *fyuA*, *kpsMTIII*	This study
405	-	NA	SP (1)	2008	C	Urine	CTX-M-15	Ak, Cp, Gm, Na, Tb, Ts	*fimH*, *fyuA*	[27]
964	-	405_7 (III)	NW (1)	2003	H	Respiratory	CTX-M-15	Cm, CpGm, Km, Na, Nf, Sm, Su, Tb, Te, Tp, Ts,	*fimH*, *traT*, *sat*, *fyuA*, *papEF*	[12]

^a^*H* Hospitalized humans (obtained from representative outbreaks), *C* Community acquired infections (obtained from representative outbreaks), *F* Healthy humans (feces), *A* Animals, *S* Ready-to-eat salads. ^b^ Variability among isolates is represented in parenthesis. ^c^Isolates identified as biotype A, ^d^Isolates identified as biotype B; ^e^Isolates identified as biotype C. ^f^ Isolate considered ExPEC. *ND* Not determined, *NA*, Not applicable, *Ak* Amikacin, *Cm* Chloramphenicol, *Cp* Ciprofloxacin, *Gm* Gentamicin, *Km* Kanamycin, *Na* Nalidixic acid, *Nt* Netilmicin, *Nf* Nitrofurantoin, *Sm* Streptomycin, *Su* Sulphonamides, *Tb* Tobramycin, *Te* Tetracyclin, *Tp* Trimethoprim, *Ts* Trimethoprim-Sulfamethoxazole, Definitions: *fimH* (type 1 fimbriae), *papA* (P fimbriae major subunit, pyelonephritis-associated), *papC* (P fimbriae assembly), *papEF* (P fimbriae minor tip pilins), *papG* allele I (papG variant), *papG* allele II (papG variant, pyelonephritis-associated), *papG* allele III (P fimbriae adhesion, cystitis-associated), *sfa*/*focDE* (S and F1C fimbriae), *bmaE* (Blood group M-specific adhesin), *gafD* (glucosamine-specific adhesin), *iha* (iron-regulated-gene-homologue adhesion), *sat* (secreted autotransporter toxin), *tsh* (serine protease autotransporter), *fyuA* (yersiniabactin receptor) *iutA* (ferric aerobactin receptor), *iroN* (catecholate siderophore receptor), *ireA* (Iron-regulated element ), *kpsMT*II (group II capsular polysaccharide), *kpsMTII* K1 (variant K1), *kpsMTII* K5 (variant K5), *kpsMT*III (group III capsular polysaccharide), *traT* (serum survival associated), *iss* (increased serum survival), *usp* (uropathogenic-specific protein), *ompT* (outer membrane protease), *malX* (pathogenicity-associated island marker.

### Clonal diversity

Relatedness among isolates was established by *Xba*I-pulsed-field gel electrophoresis (PFGE), multi-locus sequence typing (MLST, http://mlst.ucc.ie/mlst/dbs/Ecoli), and identification of *E*. *coli* phylogenetic groups and serogroups by PCR [28]. Isolates exhibiting ≥85% homology were considered to belong to the same PFGE-type. *Xba*I-profiles were compared using InfoQuest^™^ FP version 5.4 software (BioRad Laboratories), by applying the UPGMA algorithm based on the Dice coefficient (1.0% band tolerance; 1.0% optimization).

### Virulence genes profile

Screening of 38 virulence factors (VFs) including adhesins, toxins, siderophores, polysaccharide coatings and others (*malX*, *usp*, *ibeA*, *iss*, *tsh*) presumptively associated with ExPEC isolates was performed by PCR as previously described [8,28]. The Fisher’s exact test was used for each comparison, a *p* value <0.05 being considered to reveal significant differences. A strain satisfied the criteria for being ExPEC if it carried two or more of the following genes: *papA*, *papC*, *sfa*/*focDE*, *afa*/*draBC*, *iutA* and *kpsMII*[8].

### Adhesion and biofilm-producing assays

The ability of D-*E*.*coli* strains to *in vitro* adhere was investigated by a modified quantitative biofilm production assay, as previously described [28]. The *E*. *coli* strain CFT073 and the culture medium supplemented with 1% (v/v) glucose were used as positive and negative controls, respectively. Assays were performed in quintuplicate and repeated at least 4 times. The cut-off optical density (ODc) was defined as three standard deviations above the mean OD of the negative control (culture medium), and strains were classified as non-adherent (OD ≤ ODc), weakly adherent (ODc < OD ≤ 2 × ODc), moderately adherent (2 × ODc < OD ≤ 4 × ODc), or strongly adherent (OD > 4 × ODc). The ultrastructural analysis of biofilm was performed by a Field Emission Scanning Electron Microscope (FESEM) (Zeiss, Germany). Briefly, adjusted inocula (200 μl, 0.5 McF) of each strain diluted with 1.8 ml of fresh LB supplemented with 1% (v/v) glucose were added to 24-well plates with round glass coverslips (1 cm diameter) put into each well and incubated at 37°C for 24 h. The content of each well was removed and the round coverslips were washed with PBS (1%) twice. Biofilms grown on coverslips were fixed with 2,5% glutaraldehyde in Na-cacodylate 0,1 M (pH 7.4) buffer solution (AppliChem, Germany) for 2 h at room temperature. Following three washing steps with the same buffer solution, samples were dehydrated through graded ethanol (30°, 50°, 70°, 85°, 95°, 100°) and dried with hexamethyldisilazane (Alfa Aesar, USA) for 1 h30'. Samples were air dried overnight and coated by sputtering with a gold target [19].

## Results and discussion

### Diversity among clonal groups of *E*. *coli* phylogroup D

Isolates belonging to the three analysed STs exhibited inter and intraclonal variability regarding the VF profile and the ability to form biofilm. On the basis of their virulence scores, all ST69 (n = 13/13; median = 14/range = 9-15) and all ST393 (n = 11/11; median = 14/range = 8-15), and only sporadic ST405 (n = 2/11; median = 6/range = 2-14) isolates were classified as ExPEC (Table 2). While most ST69 and ST393 carried *pap* alleles (*papA*, *papC*, *papEF*, *papG II*), *iha*, *kpsMTII*-K5 and *ompT*, ST405 isolates frequently contained *fyuA*, *malX* and *traT*, suggesting the presence of different genomic islands among *E*. *coli* phylogroup D isolates.

**Table 2:** **Virulence gene profiles of phylogenetic group D *E*. *coli *clonal groups**

**Virulence genes**^**a**^		**N° of isolates (%)**			***P *value**^**a**^		
		**ST69 (n = 13)**	**ST393 (n = 11)**	**ST405 (n = 11)**	**ST69 *vs *ST393**	**ST69 *vs *ST405**	**ST393 *vs *ST405**
**Adhesins**							
	*fimH*	13 (100%)	11 (92%)	9 (82%)	0.480	0.199	0.590
	*papA*	11 (85%)	8 (67%)	0 (0%)	0.378	**0.000**	**0.001**
	*papC*	12 (92%)	10 (83%)	0 (0%)	0.593	**0.000**	**0.000**
	*papEF*	12 (92%)	9 (75%)	2(18%)	0.322	**0.001**	**0.012**
	*papG* allele I	0 (0%)	1 (8%)	0 (0%)	0.480	-	1.000
	*papG* allele II	9 (69%)	10 (83%)	0 (0%)	0.645	**0.001**	**0.000**
	*papG* allele III	9 (69%)	2 (17%)	1 (9%)	**0.015**	**0.005**	1.000
	*bmaE*	2 (15%)	0 (0%)	0 (0%)	0.480	0.482	-
	*gafD*	2 (15%)	0 (0%)	0 (0%)	0.480	0.482	-
	*iha*	10 (77%)	10 (83%)	2 (18%)	1.000	**0.012**	**0.003**
**Toxins**							
	*sat*	10 (77%)	9 (75%)	6 (55%)	1.000	0.390	0.400
	*tsh*	1 (8%)	7 (58%)	3 (27%)	**0.011**	0.300	0.214
**Siderophores**							
	*fyuA*	8 (62%)	8 (67%)	11 (100%)	1.000	**0.041**	0.093
	*iutA*	11 (85%)	11 (92%)	6 (55%)	1.000	0.182	0.069
	*iroN*	5 (39%)	1 (8%)	1 (9%)	0.160	0.166	1.000
	*ireA*	2 (15%)	0 (0%)	1 (9%)	0.480	1.000	1.000
**Capsule**							
	*kspMT* II	12 (92%)	11 (100%)	2 (18%)	1.000	**0.001**	**0.000**
	*kpsMT* III	0 (0%)	0 (0%)	5 (46%)	-	**0.011**	**0.014**
	K1	0 (0%)	4 (33%)	0 (0%)	**0.039**	-	0.093
	K5	12 (92%)	11 (100%)	0 (0%)	1.000	**0.000**	**0.000**
**Protectins**							
	*traT*	13 (100%)	3 (25%)	10 (91%)	**0.000**	0.458	**0.003**
	*iss*	5 (39%)	6 (50%)	3 (27%)	0.695	0.679	0.400
**Miscellaneous**							
	*usp*	1 (8%)	0 (0%)	0 (0%)	1.000	1.000	-
	*ompT*	12 (92%)	6 (50%)	0 (0%)	**0.030**	**0.000**	**0.014**
	*malX* (PAI)	0 (0%)	1 (8%)	7 (64%)	0.480	**0.001**	**0.009**
ExPEC status^b^		12 (100%)	11 (100%)	2 (18%)	-	**0.000**	**0.000**
Virulence score		13.23 (± 1.641)	11.67 (± 3.576)	6.27 (± 3.197)	1.000	**0.007**	0.053
Range		9 – 15	8 – 15	2 – 14	-	-	-

^a^*p* values (Fisher’s exact test) are shown in bold when *p* < 0.05. ^b^ ExPEC status defined by the presence of two or more among *papA*, *papC*, *sfa*/*foc*, *afa*/*draBC*, *iutA* and *kpsMTII*, as suggested [8].

**Figure 1:**
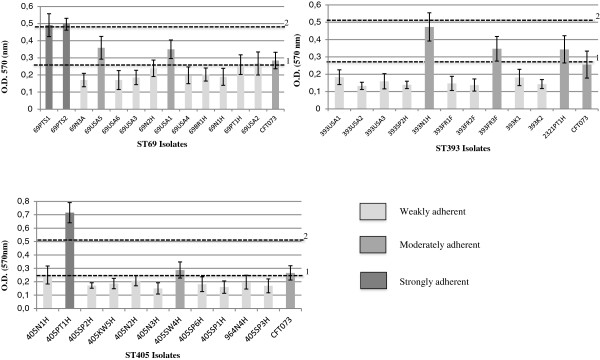
**Quantitative biofilm-producing assay.** The vertical axis represents the median optical density (OD) of at least 15 replicas of each isolate, determined at 570 nm. *E*. *coli* CFT073 was used as a positive control. Horizontal dotted lines represent the cut-off value between weakly adherent (light gray) and moderately adherent (gray) (1) and strongly adherent strains (dark grey) (2).

**Figure 2:**
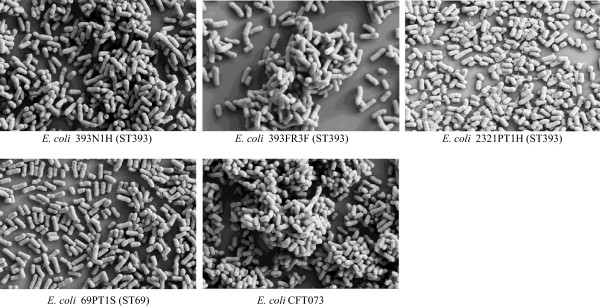
**Biofilms of strongly and moderately adherent *E*. *coli *strains.** FESEM micrographs of biofilm-growing *E*. *coli* strains were obtained at a magnification of 10.000 x using an EHT = 5.00 kV.

Most of the isolates exhibited a weak adherence ability to abiotic surfaces (9 ST69, 8 ST393, 9 ST405; 0.13 < OD < 0.27) while a few strains were classified as moderately adherent (3 ST393, 2 ST69 and 1 ST405; 0.29 < OD < 0.47) or strongly adherent (2 ST69, 1 ST405; 0.49 < O.D < 0.71) (Figure 1), and were considered as presumptive biofilm producers. Among all the strains resulting to be moderately or strongly adherent, FESEM observations revealed the presence of aggregates and EPS matrix, both compatible with a biofilm development, only in two ST69 (69PT1S, 69PT2S) and three ST393 (393FR3F, 393N1H, 2321PT1H) isolates (Figure 2). These isolates corresponded to diverse clonal variants exhibiting variable virulence gene profiles, preventing from establishing a link between this phenotype and a given virulence gene or virulence gene profile.

The presence of a characteristic virulence gene profile for isolates of different *E*. *coli* clonal groups confirms results obtained in previous studies [5,8]. However, small differences in the virulence profile observed among closely related isolates might be explained by the plasticity of the genomic islands where these genes are commonly clustered [29]. Most of the isolates were associated with extraintestinal infections (n = 25/35; 71%), including the non-ExPEC ST405 isolates. The lack of correspondence between ExPEC status and the ability to cause extraintestinal disease further suggests that other non-explored virulence factors might influence their pathogenicity [30]. Our results indicate that biofilm production seems not to be directly related with their epidemiological success, as already observed for the pandemic ST131 *E*. *coli* clone [28]. Moreover, when observed in particular strains, this feature could not be linked to a specific virulence gene or virulence profile.

### Intraclonal diversity of ST69 isolates

Thirteen isolates corresponding to 7 PFGE types were classified in different serogroups (O11, O17, O73, O77), and clustered in two groups on the basis of the similarity of the *Xba*I restriction profiles. Cluster I comprised closely related isolates (n = 10, 73.8% homology) causing hospital or community acquired infections that exhibited a common virulence gene profile (80%, *fimH*-*iha*-*iutA*-*kpsMTII*-*K5*-*traT*-*sat*-*ompT*-*papA*-*papEF*-*papGII*-*papC*). Cluster II (n = 3, 71.8% homology) included two indistinguishable isolates recovered from different samples of ready-to-eat salads in Portugal and from poultry meat in Norway. They differ in the presence of *iroN*, *iss*, *bmaE* (n = 2/3) and *gafD* (n = 2/3), and the lack of *iha*, *sat* and *papGII*, observed for isolates of cluster I. All ST69 isolates exhibited resistance to streptomycin and trimethoprim-sulfamethoxazole, and they were frequently resistant to tetracycline (85%), and to chloramphenicol (46%). None of the isolates produced ESBL, but one encoded CMY-2.

Isolates belonging to cluster I seem to have been circulating among different continents since at least 1999, as reflects this and other studies [31-33]. Despite of the small sample analysed, differences among ST69 isolates from human and non-human origins suggest independent evolution of particular *E*. *coli* variants in different hosts.

### Intraclonal diversity of ST393 isolates

These isolates corresponded to serogroups O15 (n = 9) or O25 (n = 2, one of them corresponding to ST2321, a single locus variant of ST393), and they mainly were biotype C (non-lactose fermenters and maltose fermenters; n = 7, 58.3%), which seem to be more commonly observed than those of biotype A (lactose and maltose fermenters) [4,6,34]. Most isolates analysed (n = 9/75%) were recovered from patients and healthy individuals in France, Spain, Korea and the USA and shared a pool of ten virulence genes (*fimH*-*iha*-*iutA*-*kpsMTII*-*K5*-*sat*-*papA*-*papEF*-*papGII*-*papC*) (Table 1). The ST2321 isolate belonged to O25 serotype and shared eight out of the ten frequent VFs, suggesting a common origin. Most isolates were resistant to trimethoprim-sulfamethoxazole (91%), streptomycin (91%), ciprofloxacin (82%), tetracycline (73%) and nalidixic acid (73%). Resistance against kanamycin (64%), gentamicin (36%), tobramycin (36%), netilmicin (36%) or chloramphenicol (27%) was also observed. ESBL or AmpC production was sporadically detected (1 CTX-M-14, 1 TEM-like and 1 CMY-2).

The study highlights the spread of ST393 isolates of biotype C with highly similar virulence gene profile in different continents over almost three decades, supporting previous observations in specific countries [5,8]. Unfortunately, clonal relatedness among different strains could not be analysed due to the spontaneous lysis of DNA, also reported by other groups [6,34].

### Intraclonal diversity of ST405 isolates

Isolates of this clonal complex (n = 11, 6 PFGE types) were recovered from human infections (82% hospital, 18% community), and exhibited a common virulence profile (*fimH*-*traT*-*fyuA*-*malX*, n = 6, 55%) (Table 1). Most isolates belonging to cluster I (n = 6, 2 ExPEC; 77% homology) identified in hospitalized patients from Portugal, Spain, Norway and Kuwait contained additionally *iutA* and *sat* (n = 5/6, 83%) whereas cluster II (n = 3 from Spain and Switzerland; 80% homology) showed consistently *kpsMTIII* but not *iutA* and *sat*. Cluster III comprised only one isolate from Norway corresponding to a single locus variant of ST405 (ST964). ST405 isolates were commonly resistant to streptomycin, sulphonamides, trimethoprim (91% each), kanamycin, tetracycline, nalidixic acid (82% each), gentamicin (73%), tobramycin (64%), ciprofloxacin (45%) and chloramphenicol (45%) (Table 1).

These results suggest that several ST405 variants seem to be circulating in distinct countries. In contrast with ST69 and ST393, isolates frequently produced either ESBLs (mostly CTX-M-15, but also CTX-M-3, CTX-M-14, TEM-24 or TEM-52) or AmpC (CMY-2) enzymes, which might have facilitated the selection and successful spread of diverse ST405 variants [2,13,14,35].

## Conclusion

Factors responsible for the increased ability of particular *E*. *coli* clones to successfully spread and persist are poorly understood, and our work represents one of the few studies exploring the phenotypic traits involved in the increased epidemicity of emerging antibiotic resistant *E*. *coli* clonal groups [28,36]. The results highlight the inter and intraclonal diversity of *E*. *coli* clones of phylogroup D and further suggest the circulation of highly transmissible ST69, ST393 and ST405 variants, some of them being particularly widespread in different geographic areas and settings. The lack of association between the ability to produce biofilm exhibited by a few strains and specific virulence gene or virulence gene profiles points out the need to further explore factors involved in the selection of particular epidemic variants with enhanced ability to colonize and persist for extended periods of time.

## Competing interests

The authors declare that they have no competing interests.

## Authors’ contribution

AN was responsible for study conception and design, data acquisition and analysis and drafted the manuscript. LP participated in the conception and design, analysis of data and preparation of the manuscript. CV, JP and CM contributed with data acquisition and analysis. TC and GD were implicated in data analysis and preparation of the manuscript. All authors read and approved the final manuscript.
